# Greater Numbers of Chews and Bites and Slow External Rhythmic Stimulation Prolong Meal Duration in Healthy Subjects

**DOI:** 10.3390/nu17060962

**Published:** 2025-03-10

**Authors:** Megumi Aoshima, Kanako Deguchi, Risako Yamamoto-Wada, Chihiro Ushiroda, Eri Hiraiwa, Miyuki Yokoi, Chisato Ono, Mitsuyoshi Yoshida, Katsumi Iizuka

**Affiliations:** 1 Faculty of Medicine, Fujita Health University, Toyoake 470-1192, Japan; 51021001@fujita-hu.ac.jp (M.A.); 51022099@fujita-hu.ac.jp (E.H.); 2 Department of Clinical Nutrition, Fujita Health University, Toyoake 470-1192, Japan; kanasakuran@gmail.com (K.D.); risako.wada@fujita-hu.ac.jp (R.Y.-W.); chihiro.ushiroda@fujita-hu.ac.jp (C.U.); 3 Department of Dentistry and Oral-Maxillofacial Surgery, Fujita Health University, Toyoake 470-1192, Japan; miyuki.yokoi@fujita-hu.ac.jp (M.Y.); mitsuyoshi.yoshida@fujita-hu.ac.jp (M.Y.); 4 Department of Medical Technology, Fujita Health University Haneda Clinic, Tokyo 144-0041, Japan; chisato.ono@fujita-hu.ac.jp; 5 Food and Nutrition Service Department, Fujita Health University Hospital, Toyoake 470-1192, Japan

**Keywords:** meal duration, number of chews, number of bites, chewing tempo

## Abstract

Background/Aim: Slow eating is recommended for obese individuals. We aimed to determine the associations between meal duration and various factors (sex, numbers of chews and bites, eating tempo (including forced rhythm with a metronome) and BMI). Methods: Using a test meal (a quarter slice of pizza), we tested the sex difference of the meal duration, numbers of chews and bites, and eating tempo for thirty three healthy subjects (M: 15; F: 18) aged 37.2 ± 11.1 years via unpaired *t* tests. Next, factors influencing meal duration were identified via multivariate analysis (adjusted for sex), with meal duration as the dependent variable. Results: The meal duration and numbers of chews and bites differed significantly between sexes (63.1 ± 20.7 vs. 87.4 ± 22.8, *p* = 0.003; 80.3 ± 28.7 vs. 107.0 ± 36.1, *p* = 0.02; 2.1 ± 1.1 vs. 4.5 ± 2.6, *p* = 0.001, respectively), but the chewing tempo was similar (*p* = 0.32). Meal duration was associated with the number of chews (β = 0.6 [0.4, 0.7], *p* < 0.001) and bites (5.8 [2.5, 9.2], *p* = 0.001) but not with BMI (*p* = 0.52) or chewing tempo (*p* = 0.99). Finally, when a metronome was used to force rhythmic stimulation (0/40/80/160 bpm), compared with 0 bpm, slow stimulation (40 bpm) resulted in increased meal duration (mean difference [95% CI] = −47.0 [−66.4, −27.7], *p* < 0.0001), chews (−28.6 [−44.5, 12.8], *p* = 0.0003), and bites (−4.9 [−7.9, 1.9] *p* = 0.001) and delayed tempo (10.4 [4.5, 16.3], *p* = 0.0004). Conclusions: Meal duration was positively associated with the numbers of chews and bites and negatively associated with chewing tempo. Thus, increasing the numbers of bites and chews and slowing the eating tempo may prolong meal duration.

## 1. Introduction

Obesity is a medical condition involving the accumulation of excess body fat and is associated with diseases such as fatty liver, diabetes mellitus, dyslipidemia, and cardiovascular diseases [1,2]. As factors causing obesity, the dysfunction of the reward brain system is known to lead to overeating. Namely, sugar and fat excessively stimulate the brain’s reward system, thereby exceeding appetite regulation by the hypothalamus [3]. Moreover, abnormal eating behavior is known to cause obesity [4]. Moreover, eating behavior is indicated by meal duration, chewing tempo (the rate or speed of chewing), or the number of bites or chews [5,6,7,8,9]. Eating behavior influences not only satiety but also food intake and meal duration. For example, it has been reported that people with faster chewing tempos have shorter meal durations but also consume more food [5,6,7,8,9]. Furthermore, fast eaters are more prone to obesity and metabolic syndrome [10]. Although meal duration has not been quantitatively evaluated, it has been reported that the prevalence of obesity is greater among those who perceive themselves as fast eaters [8,11,12,13,14]. A meta-analysis also reported that chewing gum can suppress appetite [15]. Thus, slow eating and more chewing are recommended for obese individuals.

In Japan, the Japanese Ministry of Agriculture, Forestry and Fisheries (MAFF) website also recommends that to eat slowly, people should allow plenty of time for meals, concentrate on eating, and eat a chewy meal [16]. Various other methods can be implemented, such as thinning the seasoning or not putting the next bite of food in the mouth while the previous bite is still in the mouth. People are often surrounded by a variety of music when dining at restaurants and other places. The possibility that such outside music may influence food intake, meal satisfaction, and meal duration has been considered, but it is a controversial topic [9,10]. However, chewing tempo is essentially constant because of the presence of a rhythm generator in the central nervous system [17,18]. Therefore, we were interested in whether external rhythmic stimuli could influence the tempo of eating.

Similar to sex differences in the frequency of obesity, eating behavior also differs between men and women. For example, there is a sex difference in food preferences between males and females [19,20,21,22,23]. Our previous studies revealed that men consume meat more frequently than women do and women consume fish more frequently than men do. Moreover, eating behavior also differs by sex [20]. Empirically, men have shorter meal durations, and women have longer meal durations. In Japan, there is a term called “ochobo-guchi”, which means a small, cute mouth (usually seen in females), and it is thought that the number of chews and how long one holds food in their mouth may also differ by sex, but there is not much evidence to link these factors together conclusively. Thus, nutrition guidance on how to eat slowly is based on empirical reasoning and lacks objective evidences on what factors shape eating behavior and lead to slow eating in Japan.

Providing nutritional guidance for individuals with obesity is thought to result in a slower chewing tempo, thereby lengthening meal duration and, in turn, reducing the amount of food consumed. Therefore, analysis of factors influencing parameters such as eating tempo, meal duration, and the numbers of chews and bites is important for obesity treatment. The aim of this study was to clarify the associations between meal duration and various factors (sex, numbers of chews and bites, chewing tempo (including forced rhythm with a metronome) and BMI). First, we identified sex differences in meal duration, number of chews, number of bites, and chewing tempo. Next, we determined the effects of the number of chews, number of bites, chewing tempo, and body mass index (BMI) on meal duration when adjusted for sex. Finally, we examined whether the eating tempo, meal duration, number of chews, and number of bites are affected by forced rhythmic stimulation with a metronome. This study revealed that meal duration is related to the numbers of chews and bites, regardless of sex, and that meal duration can be significantly prolonged by slowing the chewing tempo. The results of this study provide important evidence to support the implementation of a prolonged meal duration.

## 2. Materials and Methods

### 2.1. Subjects

This study was a prospective intervention trial in which 33 subjects aged 20 to 65 years old (male: *n* = 15 and female: *n* = 18) were recruited from medical students and faculty and staff of Fujita Health University, and it was conducted from 9 September 2024 to 12 November 2024. Subjects with diabetes, hypertension, other ambulatory diseases, or those deemed by the physician to be unsuitable for participation in this study were excluded. The study was conducted according to the principles of the Declaration of Helsinki and was approved by the Research Ethics Committee of Fujita Health University (approval number HM24-004 (approval date: 18 June 2024)). All patients provided written, informed consent before enrollment in the study. This study was registered with UMIN Clinical Trial Registry (UMIN000056594).

### 2.2. Measurement of Meal Duration, Numbers of Chews and Bites, and Eating Tempo

Meal duration was measured by pressing a stopwatch at the beginning and end of the meal. The bitescan^TM^ (Sharp Inc., Sakai, Osaka, Japan) is a machine that measures the number of chews, number of bites, and chewing tempo [24,25]. Calibration was done with bite-sized gummies. The skin behind the auricle moves as the jaw moves; the distance sensor in bitescan senses these minute changes. The number of chews and the chewing tempo are determined by a proprietary algorithm.

### 2.3. BDHQ (Brief-Type Self-Administered Diet History Questionnaire)

The BDHQ (brief-type self-administered diet history questionnaire) is one of the self-administered diet history questionnaires used in Japan [26,27]. The BDHQ requires 20 min to complete and consists of the following five sections: (i) intake frequency of 46 food and nonalcoholic beverage items; (ii) daily intake of rice, including the type of rice (refined or unrefined) and miso soup; (iii) frequency of alcoholic beverage consumption and amount consumed for five types of alcoholic beverages; (iv) usual cooking methods; and (v) general dietary behavior [26,27]. On the basis of the answers to the questions regarding the meals that the participants ate during the preceding month, the results summarize the intake of approximately 100 nutrients and 58 food items.

### 2.4. Handgrip Strength and Five-Times Sit-to-Stand Test

The five Times Sit to Stand Test (5X Sit-To-Stand Test) is commonly abbreviated as 5XSST. It’s used to assess functional lower limbs strength, transitional movements, balance, and fall risk in older adults. Hand grip strength is an indicator of upper body strength but is also used as an indicator of overall muscle strength in population studies. Grip strength was used for the dominant hand.

### 2.5. Experiments

**Figure 1:**
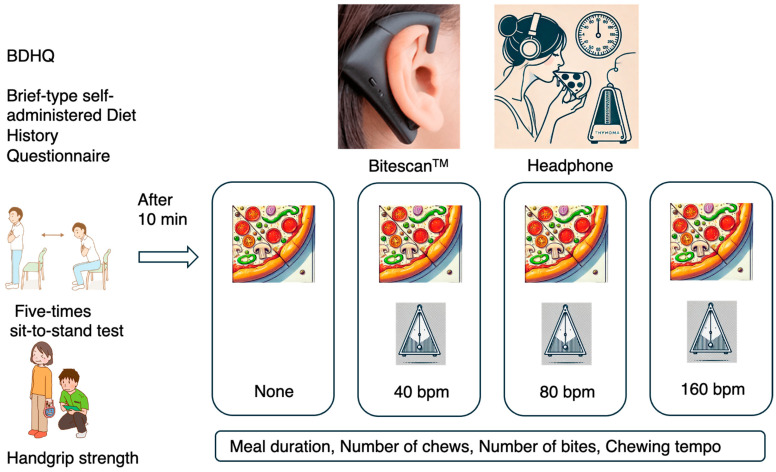
Overview of experimental process.

After ensuring that at least 4 h had passed since the subjects had breakfast, their weight and handgrip strength were measured, they performed the five-times sit-to-stand test, and, 10 min later, they began the meal measurement experiment. After putting on headphones, the subjects first ate a quarter of a slice of pizza (317 kcal (protein 13.0 g: fat 12.6 g: carbohydrate 38 g) per 1 sheet (117 g, 8 inches in diameter); Microwave Mix Pizza, Maruha Nichiro, Tokyo, Japan), and the meal duration, number of chews, average eating tempo, and number of bites were measured; after 1 min, they were instructed to eat a second quarter of a slice of pizza at a metronome tempo of 40 bpm. After 30 s, the third quarter of a slice of pizza (1/4 sheet) was eaten to a metronome tempo of 80 bpm, and the meal duration and number of chews were measured. After 30 s, the fourth quarter of a slice of pizza (1/4 sheet) was eaten to a metronome tempo of 80 bpm, and the meal duration and number of chews were measured (Figure 1).

The study was conducted on 33 healthy students or employees between the ages of 20–65 years old with no underlying medical conditions. First, they wore headphones to block out sounds from the outside world, then ate a quarter slice of pizza, following the metronome rhythm (40 bpm, 80 bpm, 160 bpm), and the duration of each meal, number of chews, mouthfuls, and chewing tempo were measured using bite scans.

### 2.6. Statistics

Because this was an exploratory study, no sample size was set. The values are expressed as the means ± standard deviations (SDs). Comparisons between men and women regarding age, BMI, handgrip strength, five-time chair stand test performance, the number of chews, the chewing tempo, the number of bites, and the food intake frequency survey results (total energy (kcal), protein (g), fat (g), carbohydrate (g), and dietary fiber (g) intake) were conducted via a *t* test (two-tailed), with *p* < 0.05 considered to indicate statistical significance. Next, a multivariate linear regression analysis was performed with meal duration as the dependent variable and the number of chews, mean tempo, number of bites, BMI, and five-times sit-to-stand test as the independent variables adjusted for sex.

A multivariate linear regression analysis was also performed with meal duration as the dependent variable and total energy intake, protein intake, fat intake, carbohydrate intake, or dietary fiber intake as the independent variables adjusted for sex. Finally, among the metronome 0 bpm, 40 bpm, 80 bpm, and 160 bpm groups, statistical analysis was performed via one-way ANOVA followed by Tukey post hoc tests. A *p* value < 0.05 was considered to indicate statistical significance. GraphPad Prism version 10 (GraphPad Software Inc., San Diego, CA, USA) was used as the statistical software.

## 3. Results

### 3.1. Background of the Subjects in This Study

Thirty three healthy subjects participated in this study. None of the subjects were excluded. The age of the subjects in this study was 37.2 ± 11.1 years (y.o.) (M: 37.1 ± 11.3 vs. F: 37.3 ± 11.2, *p* = 0.95) (Table 1). The BMI of the subjects in this study was 23.0 ± 3.2 (M: 24.1 ± 3.1 vs. F: 22.0 ± 3.0, *p* = 0.07). Thus, the age and BMI were similar between males and females.

While handgrip strength was greater in males (M: 41.0 ± 5.2 vs. F: 27.2 ± 5.0, *p* < 0.001), there was no difference between males and females in the five-times sit-to-stand test (M: 6.9 ± 1.2 vs. F: 7.0 ± 1.0, *p* = 0.83). The meal durations were longer in females than in males (M: 63.1 ± 20.7 vs. F: 87.4 ± 22.8, *p* = 0.003). The total numbers of chews (M: 80.3 ± 28.7 vs. F: 107.0 ± 36.1, *p* = 0.02) and bites (M: 2.1 ± 1.1 vs. F: 4.5 ± 2.6, *p* = 0.001) were also greater in females than in males. In contrast, the chewing tempo and total energy, protein, fat, carbohydrate, and dietary fiber intakes were similar (83.6 ± 9.3 vs. 79.9 ± 11.9, *p* = 0.32; 1626.7 ± 508.3 vs. 1401.7 ± 342.2, *p* = 0.14; 55.8 ± 15.9 vs. 52.7 ± 19.9, *p* = 0.63; 47.5 ± 16.1 vs. 46.7 ± 15.4, *p* = 0.88; 215.2 ± 81.9 vs. 177.8 ± 38.7, *p* = 0.095; 8.5 ± 3.3 vs. 8.1 ± 3.3, *p* = 0.76, respectively). Thus, the meal duration, the total numbers of chews and bites, and handgrip strength differed between males and females.

### 3.2. The Meal Duration Was Associated with the Numbers of Chews and Bites but Not the Average Eating Tempo

According to the above data, the meal duration differed between males and females. We subsequently examined the associations between the meal durations and several factors adjusted for sex. The meal duration was associated with the numbers of chews (β [95% CI] = 0.6 [0.4, 0.7], *p* < 0.001) and bites (β [95% CI] = 5.8 [2.5, 9.2], *p* = 0.001). In contrast, the meal duration was not significantly associated with the chewing tempo (−0.002 [−0.8, 0.8], *p* = 0.995), BMI (−0.9 [−3.5, 1.8], *p* = 0.52) or the number of repetitions in the five-times sit-to-stand test (3.4 [−4.0, 10.8], *p* = 0.36) (Table 2).

The total energy and nutrient intake and meal duration were not significantly associated with total energy (0.01 [−0.007, 0.03], *p* = 0.21), protein (0.4 [−0.05, 0.8], *p* = 0.08), fat (0.42 [−0.08, 0.91], *p* = 0.1), carbohydrate (0.06 [−0.07, 0.2], *p* = 0.36), or dietary fiber (1.4 [−1.0, 3.9], *p* = 0.24) intake (Table 3).

Thus, the meal duration was significantly associated with only the numbers of chews and bites but not the average chewing tempo.

### 3.3. The Slow Rhythm Produced by the Metronome More Potently Prolonged the Meal Duration and Increased the Numbers of Chews and Bites by Decreasing the Chewing Tempo

To better understand whether eating to the rhythm of a metronome could regulate meal duration and chewing frequency. Since the chewing tempo was approximately 80 bpm, we set 80 bpm as the standard tempo and examined the effects of eating at 40 bpm and 160 bpm.

**Figure 2:**
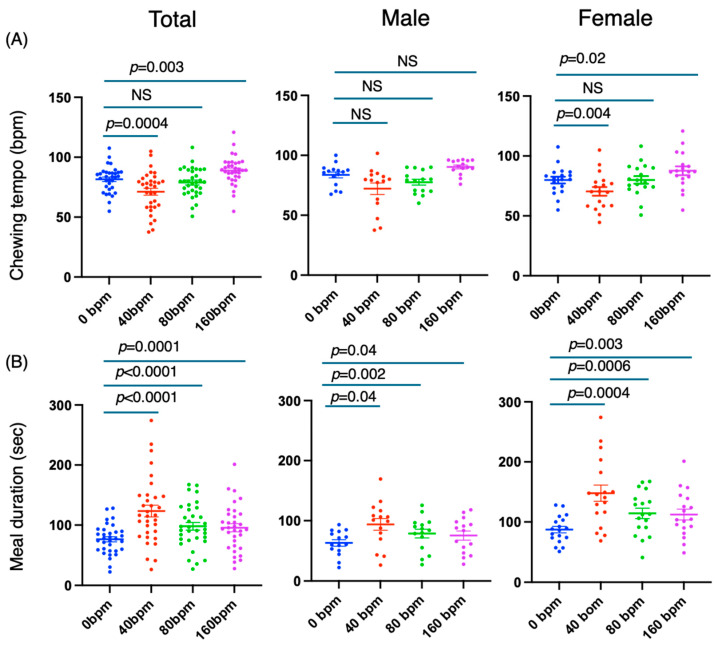

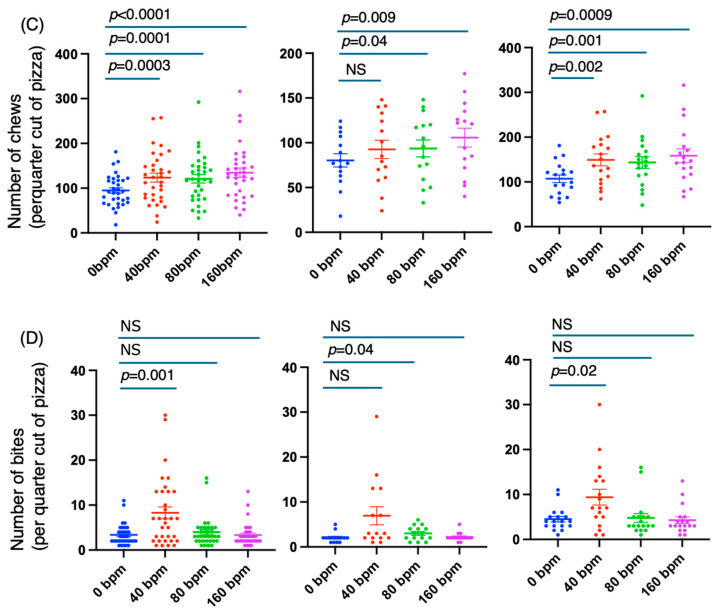
Effects of external stimuli by metronome on meal duration, number of chews, number of bites, and chewing tempo. (**A**) chewing tempo, (**B**) meal duration, (**C**) number of chews, (**D**) number of bites. Experimental procedure was described in Figure 1. Chewing tempo, number of chews and number of bites were measured by bitescan^TM^ (Sharp Co., Ltd., Sakai, Osaka, Japan) and meal duration was measured by stopwatch. NS = no significant.

First, regarding the chewing tempo, a significant reduction in tempo was observed at 40 bpm compared with when nothing was played (mean difference (0–40 bpm) [95% CI] = 10.39 [4.459–16.33], *p* = 0.0004) (Figure 2A). On the other hand, no significant difference was found at 80 bpm (mean difference [95% CI] = 2.7 [−2.0 to 7.4], *p* = 0.37) compared with when nothing was played. The mean difference (0–160 bpm) [95% CI] was significant (−7.2 [−12.1–2.4], *p* = 0.003), but the change was slight (Figure 2A). Sex-specific analysis revealed that the chewing tempo was affected significantly only for females (mean difference (0–40 bpm): 9.466 [3.027, 15.90], *p* = 0.004; 0–160 bpm, −7.7 [−14.0, −1.4], *p* = 0.02). Thus, the chewing tempo was more strongly affected when the metronome stimulus was 40 bpm compared to 160 bpm (Figure 2A).

Next, with respect to meal duration, at 40 bpm, meal duration was prolonged regardless of sex (mean difference [95% CI] = −47.0 [−66.4, −27.7], *p* < 0.0001). On the other hand, meal durations were also prolonged at 80 bpm and 160 bpm (mean difference: −21.7 [−30.5, −12.9], *p* < 0.0001, −19.2 [−29.3, −9.2], *p* = 0.0001) but not as much as at 40 bpm (Figure 2B). These changes were observed in both sexes.

The number of chews increased in the order of 160 bpm, 40 bpm, and 80 bpm (0 bpm–40 bpm: −28.6 [−44.5, −12.8], *p* = 0.0003; 0 bpm–80 bpm: −25.9 [−39.2, −12.6], *p* = 0.0001; 120 bpm: −39.6 [−57.7, −21.6], *p* < 0.0001) (Figure 2C). These changes were observed in both men and women.

Finally, for the number of bites, there was an increase at 40 bpm (−4.9 [−7.9, −1.9], *p* = 0.0010) and no significant difference at 80 bpm and 160 bpm (*p* = 0.25 and 0.99, respectively). The change at 40 bpm was significant only for females (0–40 bpm: male: −4.9 [−9.8, 0.02], *p* = 0.051; female: −4.9 [−9.2, −0.6], *p* = 0.02) (Figure 2D).

These results indicate that at 40 bpm, there was an increase in meal duration, an increase in the number of chews, a decrease in eating tempo, and an increase in the number of bites. However, at 160 bpm, chewing tempo, the meal duration, and number of chews changed significantly but not as significantly as the number of bites did.

## 4. Discussion

Although people with obesity are believed to have longer meal durations, this is often based on their own self-reporting and anecdotal reports, but this has not been quantified. Additionally, people are advised to slow their meal durations, but this approach is not always well founded. We aimed to determine the associations of meal duration, sex, number of chews, number of bites, and chewing tempo (including forced rhythm with a metronome) with BMI. In this study, using a test meal (pizza), we estimated the associations between the meal duration and the number of chews, number of bites, chewing tempo, muscle strength (five-times sit-to-stand test), and normal dietary conditions (total energy and major nutrients) adjusted for sex. After adjusting for sex, the duration of meals was associated with the numbers of chews and bites but not with the average eating tempo or BMI. Next, we tested the effects of the metronome-enforced eating tempo on the average eating tempo, meal duration, and the numbers of chews and bites. Slowing the rhythm of the metronome allowed for a slower eating tempo, longer meal durations, and more bites and chews. On the other hand, faster metronome tempos had a significantly different but smaller effect on chewing tempo, meal durations, and the numbers of chews and bites. Thus, meal duration is associated with the numbers of chews and bites and is less strongly associated with BMI. A slow tempo with a metronome can be used to regulate time for meals, the number of chews, and the number of bites. After adjustment for sex, meal duration was associated with the numbers of chews and bites, independent of BMI. Therefore, in addition to increasing the numbers of chews and bites, eating to a slower rhythm may help increase the meal durations.

The meal duration and numbers of chews and bites were differed by sex. Consistent with our data, self-reported results also show that men and women are more likely to eat very fast (13.3% vs. 9.2%) and fast (37.5% vs. 35.1%), respectively. It has been reported that sex hormones have an important role in food behaviors in rodents. In rodents, the meal duration in females is also shorter than that in males [28,29,30,31,32]. One reason is that estrogen inhibits food intake probably via the ventromedial hypothalamus, whereas progesterone and testosterone may stimulate appetite. In fact, males eat more than females, but gonadectomy had the opposite effect: castration of males resulted in weight loss because of decreased eating, while castration of females increased body weight due to hyperphagia. These changes were restored by testosterone and estrogen replacement, respectively [31,32]. There is also a significant sex difference in taste preference [19]. Taken together, these findings indicate that meal duration is associated with the numbers of chews and bites and that there are sex differences in meal duration and the numbers of chews and bites.

Meal duration refers to the amount of time spent eating during a single meal. In our study, there was no association between meal duration and BMI adjusted for sex. In contrast, self-reported data indicate that fast eaters have a greater increase in BMI from age 20 than slow eaters do. According to the 2009 National Health and Nutrition Survey, the higher the obesity level of both men and women is, the greater the percentage of fast eaters and the smaller the percentage of slow eaters [33]. The discrepancy in these results likely stems from self-reported diets differing from objective dietary measures. According to a report on the number of chews and meal duration, pre-World War-II Japanese people chewed 1420 times and ate for approximately 22 min, whereas current meals consist of 620 chews and 11 min, a decrease of half in both the number of chews and the meal duration [16]. One contributing factor is that, in contrast to contemporary dietary habits, pre-World War II Japanese populations predominantly consumed cereals such as wheat, root vegetables, and dehydrated foods such as koya-tofu. These findings indicate that meal content strongly influences the meal duration. Therefore, when providing nutritional guidance for individuals with obesity, it is necessary not only to recommend lengthening the duration of a meal but also to fully consider the content of the diet.

As mentioned above, eating behaviors such as meal duration and food intake are influenced by sex hormones, so it was expected that the chewing tempo would also be slower in females. However, in our data, the chewing tempo was similar between males and females. Other studies reported that there were no differences in the masticatory rhythm between males and females [34]. One of the reasons why chewing tempo does not change with sex is that there is an inherent rhythm [17,18]. The basic pattern of rhythmic jaw movements produced during mastication is generated by a neuronal network located in the brainstem and referred to as the masticatory central pattern generator (CPG) [17,18]. Moreover, we showed that, regardless of whether the metronome tempo was doubled or halved, the change in chewing tempo was minimal compared with the rhythmic changes in the metronome. Thus, the chewing tempo is constant and not significantly affected by sex.

Meal duration was significantly associated with the numbers of chews and bites but not the chewing tempo. Consistent with these findings, in a previous study, as the number of chews increased, the duration of meals increased [35]. However, slower eating has been shown to reduce food or energy intake [36,37,38]. Ferster, Nurnberger, & Levitt proposed that by reducing the chewing tempo and taking smaller bites, people with obesity reduce their food intake [9]. Consistent with these findings, we showed that using a metronome to force the eating tempo to be faster did not change the chewing tempo significantly. On the other hand, when the metronome was forced to slow, the changes in chewing tempo, meal duration, and the numbers of chews and bites were clearly greater than when the metronome was forced to speed up. Therefore, increasing the numbers of chews and bites and slowing the chewing tempo are likely to lead to longer meal durations and, in turn, may reduce food intake.

A limitation of the present study is that it is an exploratory study with a small sample size, but significant changes were still observed. The next limitation of the study is that it is not a randomized, comparative study. Since the possibility cannot be ruled out that the speed of chewing may slow down over time, it may be necessary to consider using tempos of 160 bpm, 80 bpm, and 40 bpm, in that order, instead of the other way around. However, as this study shows, the tempo of the meals remained fairly constant throughout the study period and the amount of pizza used was very small, so the effect on the results was minimal. Furthermore, since the test meal was an easy-to-eat pizza, the results need to be verified with a greater variety of items before they can be generalized to other foods. In addition, the current study included normal-weight and partially obese subjects. Future studies should clarify whether the relationship between the meal duration and the numbers of chews and bites can be generalized to foods of varying palatability. Next, we would like to clarify the relationships among meal duration, number of chews, number of bites, and amount of food consumed when the eating tempo is slowed with a metronome in obese patients, since slowing the eating tempo may also reduce the amount of food consumed in obese patients.

## 5. Conclusions

In conclusion, there was a sex difference in meal duration and the numbers of chews and bites but not in the average eating tempo. By adjusting for sex, meal duration was significantly associated with the numbers of chews and bites but not the average eating tempo or intake of various nutrients. Independent of sex, increasing the numbers of chews and bites as well as forcing individuals into a slower rhythm would lengthen meal durations in obese subjects. The chewing of chewy foods is expected to increase meal duration as well. Increasing the number of bites by eating with small mouthfuls or using a small spoon may lead to longer mealtimes. Furthermore, on the basis of the results of our metronome experiment, playing relaxing music during meals may also be effective in extending the meal duration. The results of this study should be confirmed by replicating this procedure with a variety of foods in the future, as that would make it possible to apply the results to obese patients clinically.

## Figures and Tables

**Table 1:** Background of the individuals in this study.

	Total (*n* = 33)	Male (*n* = 15)	Female (*n* = 18)	*p*
Age (years)	37.2 (11.1)	37.1 (11.3)	37.3 (11.2)	0.95
BMI (kg/m^2^)	23.0 (3.2)	24.1 (3.1)	22.0 (3.0)	0.07
Handgrip strength (kg)	33.5 (8.5)	41.0 (5.2)	27.2 (5.0)	<0.001
Five-times sit-to-stand test (s)	7.0 (1.1)	6.9 (1.2)	7.0 (1.0)	0.83
Meal duration (s)	76.3 (24.8)	63.1 (20.7)	87.4 (22.8)	0.003
Number of chews	94.8 (35.2)	80.3 (28.7)	107.0 (36.1)	0.02
Chewing Tempo (bpm)	81.6 (10.8)	83.6 (9.3)	79.9 (11.9)	0.32
Number of bites	3.4 (2.4)	2.1 (1.1)	4.5 (2.6)	0.001
Total energy (kcal)	1504.0 (427.4)	1626.7 (508.3)	1401.7 (342.2)	0.14
Protein intake (g)	54.1 (17.8)	55.8 (15.9)	52.7 (19.9)	0.63
Fat intake (g)	47.1 (15.22)	47.5 (16.1)	46.7 (15.4)	0.88
Carbohydrate intake (g)	194.8 (63.0)	215.2 (81.9)	177.8 (38.7)	0.095
Dietary fiber intake (g)	8.3 (3.2)	8.5 (3.3)	8.1 (3.3)	0.76

Data was represented as the mean (SD). Comparisons between men and women regarding age, BMI, handgrip strength, five-time chair stand test performance, the number of chews, the chewing tempo, the number of bites, and the food intake frequency survey results (total energy (kcal), protein (g), fat (g), carbohydrate (g), and dietary fiber (g) intake) were conducted via a *t* test (two-tailed), with *p* < 0.05 considered to indicate statistical significance.

**Table 2:** Multivariate linear analysis of meal duration and several factors (number of chews, eating tempo, number of bites, BMI, five-times sit-to-stand test) adjusted by sex.

Dependent Value	Meal Duration									
	Model 1		Model 2		Model 3		Model 4		Model 5	
Independent Value	*β* [95% CI]	*p*	*β* [95% CI]	*p*	*β* [95% CI]	*p*	*β* [95% CI]	*p*	*β* [95% CI]	*p*
Number of chews	0.6 [0.4, 0.7]	<0.001								
Eating tempo (bpm)			−0.002 [−0.8, 0.8]	0.99						
Number of bites					5.8 [2.5, 9.2]	0.001				
BMI (kg/m^2^)							−0.9 [−3.5, 1.8]	0.52		
Five-times sit-to-stand test (s)									3.4 [−4.0, 10.8]	0.36
Sex (M: 1, F: 0)	−9.3 [−18.5, −0.1]	0.047	−24.3 [−40.4, −8.2]	0.004	−10.1 [−25.6, 5.5]	0.2	−22.5 [−39.2, −5.9]	0.01	−24.0 [−39.6, −8.3]	0.004

Multivariate linear regression analysis was performed with meal duration as the de-pendent variable and the number of chews (Model 1), mean tempo (Model 2), number of bites (Model 3), BMI (Model 4), five-times sit-to-stand test (Model 5) as the independent variables adjusted for sex.

**Table 3:** Multivariate analysis of meal duration and several factors (energy, protein, fat, carbohydrate, dietary fiber) adjusted by sex.

Dependent Value	Meal Duration									
	Model 1		Model 2		Model 3		Model 4		Model 5	
Independent Value	*β* [95% CI]	*p*	*β* [95% CI]	*p*	*β* [95% CI]	*p*	*β* [95% CI]	*p*	*β* [95% CI]	*p*
Energy (kcal)	0.01 [−0.007, 0.03]	0.21								
Protein (g)			0.4 [−0.05, 0.8]	0.08						
Fat (g)					0.4 [−0.08, 0.9]	0.1				
Carbo-hydrate (g)							0.06 [−0.07, 0.2]	0.36		
Dietary fiber (g)									1.4 [−1.0, 3.9]	0.24
Sex (M: 1, F: 0)	26.9 [10.9, 42.9]	0.002	25.4 [10.3, 40.6]	0.002	24.6 [9.5, 39.8]	0	26.4 [10.1, 42.8]	0.002	24.8 [9.3, 40.3]	0.003

Multivariate linear regression analysis was performed with meal duration as the de-pendent variable and total energy intake (Model 1), protein intake (Model 2), fat intake (Model 3), carbohydrate intake (Model 4), or dietary fiber intake (Model 5) as the independent variables adjusted for sex.

## Data Availability

Some or all datasets generated during and/or analyzed during the current study are not publicly available but are available from the corresponding author upon reasonable request.
